# Abnormal temporal variability of rich-club organization in three major psychiatric conditions

**DOI:** 10.3389/fpsyt.2023.1226143

**Published:** 2023-08-31

**Authors:** Meng Niu, Hanning Guo, Zhe Zhang, Yu Fu

**Affiliations:** ^1^Department of Radiology, The First Hospital of Lanzhou University, Lanzhou, China; ^2^Intelligent Imaging Medical Engineering Research Center of Gansu Province, Lanzhou, China; ^3^Accurate Image Collaborative Innovation International Science and Technology Cooperation Base of Gansu Province, Lanzhou, China; ^4^Institute of Neuroscience and Medicine, Medical Imaging Physics (INM-4), Forschungszentrum Jülich, Jülich, Germany; ^5^School of Physics, Hangzhou Normal University, Hangzhou, China; ^6^Institute of Brain Science, Hangzhou Normal University, Hangzhou, China; ^7^College of Information Science & Electronic Engineering, Zhejiang University, Hangzhou, China

**Keywords:** major psychiatric disorders, rich-club organization, temporal properties, functional connectivity, brain networks

## Abstract

**Introduction:**

Convergent evidence has demonstrated a shared rich-club reorganization across multiple major psychiatric conditions. However, previous studies assessing altered functional couplings between rich-club regions have typically focused on the mean time series from entire functional magnetic resonance imaging (fMRI) scanning session, neglecting their time-varying properties.

**Methods:**

In this study, we aim to explore the common and/or unique alterations in the temporal variability of rich-club organization among schizophrenia (SZ), bipolar disorder (BD), and attention deficit/hyperactivity disorder (ADHD). We employed a temporal rich-club (TRC) approach to quantitatively assess the propensity of well-connected nodes to form simultaneous and stable structures in a temporal network derived from resting-state fMRI data of 156 patients with major psychiatric disorders (SZ/BD/ADHD = 71/45/40) and 172 healthy controls. We executed the TRC workflow at both whole-brain and subnetwork scales across varying network sparsity, sliding window strategies, lengths and steps of sliding windows, and durations of TRC coefficients.

**Results:**

The SZ and BD groups displayed significantly decreased TRC coefficients compared to corresponding HC groups at the whole-brain scale and in most subnetworks. In contrast, the ADHD group exhibited reduced TRC coefficients in longer durations, as opposed to shorter durations, which markedly differs from the SZ and BD groups. These findings reveal both transdiagnostic and illness-specific patterns in temporal variability of rich-club organization across SZ, BD, and ADHD.

**Discussion:**

TRC may serve as an effective metric for detecting brain network disruptions in particular states, offering novel insights and potential biomarkers into the neurobiological basis underpinning the behavioral and cognitive deficits observed in these disorders.

## 1. Introduction

Mental disorders, characterized by behavioral or mental patterns causing significant distress or impairment of personal functioning, affect 1 in 8 people and 1 in 4 family worldwide (1, 2). Due to overlapping features, accurate diagnoses of mental disorders, such as schizophrenia (SZ) and bipolar disorder (BD), present challenges (3, 4). Similarly, the differential diagnosis of attention deficit hyperactivity disorder (ADHD) and BD remains difficult due to shared symptoms and high comorbidity rates (5, 6). Understanding the homogeneity and heterogeneity of various mental disorders could elucidate their neurobiological underpinnings and inform the development of targeted diagnostic and treatment strategies.

Recent advances in functional magnetic resonance imaging (fMRI) have highlighted the importance of functional connectivity (FC) in the pathophysiology of mental disorders. Abnormalities in FC have been observed in patients with mental disorders at regional, subnetwork, and whole-brain scales (7–9). Employing graph theory-based approaches, researchers have identified disturbances in brain network topology that serve as valuable classification features for distinguishing patients with mental disorders from healthy controls (10, 11). Rich-club organization, a core feature of the brain networks, has garnered increasing attention in network neuroscience research (12, 13) and has provided novel insights into mental disorders (14–16). This organization refers to a set of brain hub regions with disproportionately high number of edges and interconnections, facilitating efficient communication between brain regions. Altered rich-club organization may indicate disrupted brain function and information transmission in specific disease states (17). Moreover, rich-club organization can reflect the trade-off between costs and benefits in human brain function, indicating the efficiency of brain operation (13). However, the topological properties of rich-club organization in mental disorders remain underexplored, particularly in terms of temporality and simultaneity.

Many previous studies have constructed static functional brain networks, aggregating FC data over entire scanning sessions and failing to capture the temporal dynamics of rich-club organization (15, 16). Static networks may inaccurately represent rich-club organization, as they could include edges active at unrelated times rather than simultaneously (18, 19). Consequently, contemporary research has shifted focus to dynamic FC, capturing time-varying patterns in brain connectivity (20–22). Despite the identification of time-varying features associated with mental disorders, the temporality, simultaneity, and time span of rich-club organization interactions remain largely unexplored.

This study aims to investigate the transdiagnostic and/or illness-specific disruptions of time-varying rich-club organization properties across SZ, BD, and ADHD. We introduce a novel temporal rich-club (TRC) analysis workflow to assess the tendency of well-connected nodes to form simultaneous and stable structures in temporal networks. Our analysis encompasses whole-brain and subnetwork scales and considers various network sparsity levels, sliding window strategies, sliding window lengths, sliding window steps, and duration of TRC coefficients. We hypothesize that: (1) the novel TRC workflow will reveal altered rich-club coefficients in major psychiatric conditions, providing a more nuanced and dynamic perspective across the entire time series and (2) different major psychiatric conditions may be distinguishable based on global or local attributes of TRC coefficients.

## 2. Materials and methods

### 2.1. Participants

All participants included in this study were obtained from two open-access datasets. Specifically, the patients with SZ and corresponding heathy controls (HCs) were collected from the Center for Biomedical Research Excellence (COBRE) database.^1^ The COBRE database contains raw anatomical and functional MR data from 74 patients with SZ and 74 HCs (ages ranging from 18 to 65 in each group). In total, 71 patients with SZ and 74 HCs from the dataset were used for our subsequent experiments, as the class labels of the other three SZ participants were not provided. Similar inclusion criteria can be found in some previous studies (9, 23, 24). The BD, ADHD patients and the corresponding HCs were collected from the Consortium for Neuropsychiatric Phenomics (CNP) database^2^ (25). After quality control, age- and sex- matching, 45 BD patients, 40 ADHD patients and 98 HCs were included in subsequent analyses. Detailed demographic information can be found in Tables 1, 2.

**Table 1:** Detailed demographic information for COBRE database.

Type	SZ	HC	*p*-values
Age (years)	38.1±13.9	35.8±11.5	0.27 ^a^
Gender (males/females)	57/14	51/23	0.11 ^b^

a Two-sample *t*-test.

b Chi-square two-tailed test.

**Table 2:** Detailed demographic information for CNP database.

Type	ADHD	BD	HC	*p*-values-1	*p*-values-2
Age (years)	32.1±10.4	35.2±9.1	33.3±8.3	0.44 ^a^	0.23 ^a^
Gender (males/females)	21/19	26/19	52/46	0.95 ^b^	0.60 ^b^

a Two-sample *t*-test.

b Chi-square two-tailed test.

The *p*-values-1 are calculated based on ADHD group vs. HC group, while *p*-values-2 are calculated based on BD group vs. HC group.

### 2.2. Imaging data preprocessing

All fMRI images were preprocessed using Data Processing & Analysis for Brain Imaging (DPABI) (26). The preprocessing procedure included the removal of the first 10 volumes of functional runs due to fMRI signal instability. Slice time correction, head-motion correction, and co-registration of T1-weighted MRI images and fMRI images were performed for the remaining volumes. Subsequently, all fMRI images were normalized to Montreal Neurological Institute (MNI) space and re-sampled to 3×3×3 mm voxels. Smooth (4 mm FWHM) and band-pass filter (0.01–0.1 Hz) were applied to the images transformed to the MNI space.

To construct FC matrices, all brain images were parcellated into 160 regions by registering images to Dosenbach’s 160 atlas after data preprocessing (27). For ease of comparison, a canonical division of brain regions into cognitive systems was used (28), dividing the 160 brain regions of each participant into six functional subnetworks: visual network (VSN), sensory-motor network (SMN), dorsal attention network (DAN), ventral attention network (VAN), frontoparietal network (FPN), default mode network (DMN), and subcortical network (SBN). According to the division scheme in Yan et al. (28), there were 14, 36, 28, 32, 21, and 22 functional brain regions for DAN, DMN, FPN, SMN, VAN, and VSN, respectively. We mainly analyzed and discussed the TRC coefficients in DAN, DMN, FPN, SMN, VAN, and VSN in later sections, while the remaining seven brain regions in SBN are not included.

### 2.3. Construction of FC matrix

This study constructed two types of FC matrices: whole-brain-scale FC matrix and subnetwork-scale FC matrix. For all matrices, the level of FC between each pair of nodes (brain regions) was computed as the Pearson correlation coefficient (PCC) between their averaged regional time series. This classical method can be found in many recent studies (7, 16, 24). The sizes of whole-brain-scale FC matrix and subnetwork-scale FC matrix are represented as N×N and M×M, respectively, where N denotes the total number of brain regions of the whole brain, and M denotes the total number of brain regions in a specific subnetwork. By setting an absolute or proportional threshold (9, 10), if the connection strength (e.g., based on the correlation values of bold signals) between two brain regions is higher than the threshold (e.g., R>0.4 for PCC), then it is considered that there is an edge connection between the two brain regions (each edge corresponds to a degree).

### 2.4. Definition of temporal rich-club effect

Before introducing the temporal rich-club effect, let us review the classical rich-club effect. The rich-club effect is defined as the density of edges in the subset $S_{>k}$ of the $N_{>k}$ nodes with degree larger than *k*, that is, $\phi \left(k\right)=\frac{2E_{>k}}{N_{>k}\left(N_{>k}-1\right)}$, where $E_{>k}$ is the number of edges among $S_{>k}$. A larger ϕ(k) indicates that nodes have a disproportionately high number of edges and many edges between each other, termed the “rich-club” effect (16, 19, 29).

Many previous brain network studies of fMRI analyze the rich-club effects in an average manner (i.e., using average values across the whole time series or a duration of the time series by dividing sliding windows). However, static networks are often aggregated representations of the resulting temporal networks (30). Thus, the rich-club structures found in such static networks could be formed by edges that were active at unrelated times (18). Since the human brain is a dynamic system, analyzing the dynamic and temporal properties of rich-club organizations may better reflect the complex neuroscience mechanisms (e.g., the hub nodes in the rich-club organizations may change continuously) during a duration of time or the whole time series.

The temporal rich-club (TRC) phenomenon has been observed recently by Pedreschi et al. (19) using different levels of network datasets such as the US air transportation network dataset. Given a temporal network, the TRC quantifies whether nodes that interact with increasing numbers of other nodes tend to interact with each other simultaneously and in a stable way (19). To consider temporality, Pedreschi et al. (19) propose to define at each time t the Δ −cohesion $\epsilon_{>k}\left(t,\Delta \right)$. It represents the number $\left|E_{>k}\left(t,\Delta \right)\right|$ of ties $E_{>k}\left(t,\Delta \right)$ (between the nodes of $S_{>k}$) that remain stable over |t, t+Δ)|, normalized by its maximal possible value $N_{>k}\left(N_{>k}-1\right)/2$. Note that $\epsilon_{>k}\left(t,\Delta =1\right)$ is the instantaneous density between the nodes of $S_{>k}$. The Δ=1 in this paper refers to duration equals to one fMRI timepoint. Therefore, the TRC coefficient can be easily defined as the maximal density of temporal edges observed in a stable way for a duration *Δ* among nodes of aggregated degree larger than k:

$$M\left(k,\Delta \right)\equiv \underset{t}{\max}\epsilon_{>k}\left(t,\Delta \right)$$

where the *M*(*k*, Δ) quantifies whether the static rich-club patterns correspond to a structure that actually existed at some instant and is formed by links that appeared at unrelated times and not in a simultaneous way. A *M*(*k*, Δ) increasing with *k* denotes the TRC effects in a simultaneous way for a duration of at least Δ. Given the consideration of complex brain dynamic patterns, performing the TRC analyses at the regional scale may shed light on the understanding of functional temporality of major psychiatric groups.

### 2.5. Calculation of temporal rich-club coefficients

In this study, we performed both whole-brain-scale TRC analyses and subnetwork-scale TRC analyses. The whole-brain-scale TRC analyses of fMRIs were calculated based on the correlation matrix constructed by PCC (i.e., 160×160 in this study). To investigate the dynamic TRC relationships, we divided the BOLD signals of the whole time series into different windows (i.e., a duration of bold signals). In later sections, we set *k* as half of the number of brain regions in the whole brain or subnetworks for whole-brain-scale and subnetwork-scale TRC analyses, respectively. As there is no universally appropriate window size and step of window shifting for fMRI data, we extensively explored adopting both the non-overlapping sliding window and overlapped sliding window methods for the TRC analyses with different window sizes and sliding steps. For non-overlapping sliding window methods, we evaluated the following parameter settings: a. window size = 5 TR, sliding step = 5 TR, sparsity threshold R≥0.2; b. window size = 5 TR, sliding step = 5 TR, sparsity threshold R≥0.4; c. window size = 5 TR, sliding step = 5 TR, sparsity thresholdR≥0.6; d. window size = 5 TR, sliding step = 5 TR, sparsity threshold R≥0.8; e. window size = 10 TR, sliding step = 10 TR, sparsity threshold R≥0.2; f. window size = 10 TR, sliding step = 10 TR, sparsity threshold R≥0.4; g. window size = 10 TR, sliding step = 10 TR, sparsity threshold R≥0.6; h. window size = 10 TR, sliding step = 10 TR, sparsity threshold R≥0.8.

**Figure 1:**
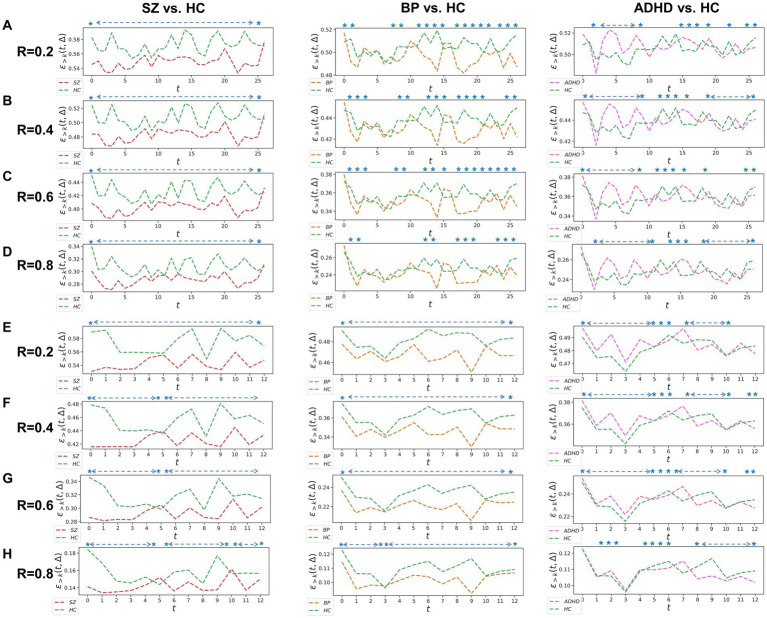
Whole-brain between-group TRC analyses for major psychiatric groups vs. corresponding HC groups on non-overlapping sliding window methods. Each *t* in all subgraphs corresponds to a sliding window. The subgraph **(A–D)** represent the whole-brain TRC coefficients of major psychiatric groups vs. HC groups based on the window size = 5 TR and sliding step = 5 TR, while the subgraph **(E–H)** represent the whole-brain TRC coefficients of major psychiatric groups vs. HC groups based on the window size = 10 TR and sliding step = 10 TR. All of the asterisks (*) denote significant between-group differences in this Δ = 1 based on *t*-tests.

**Figure 2:**
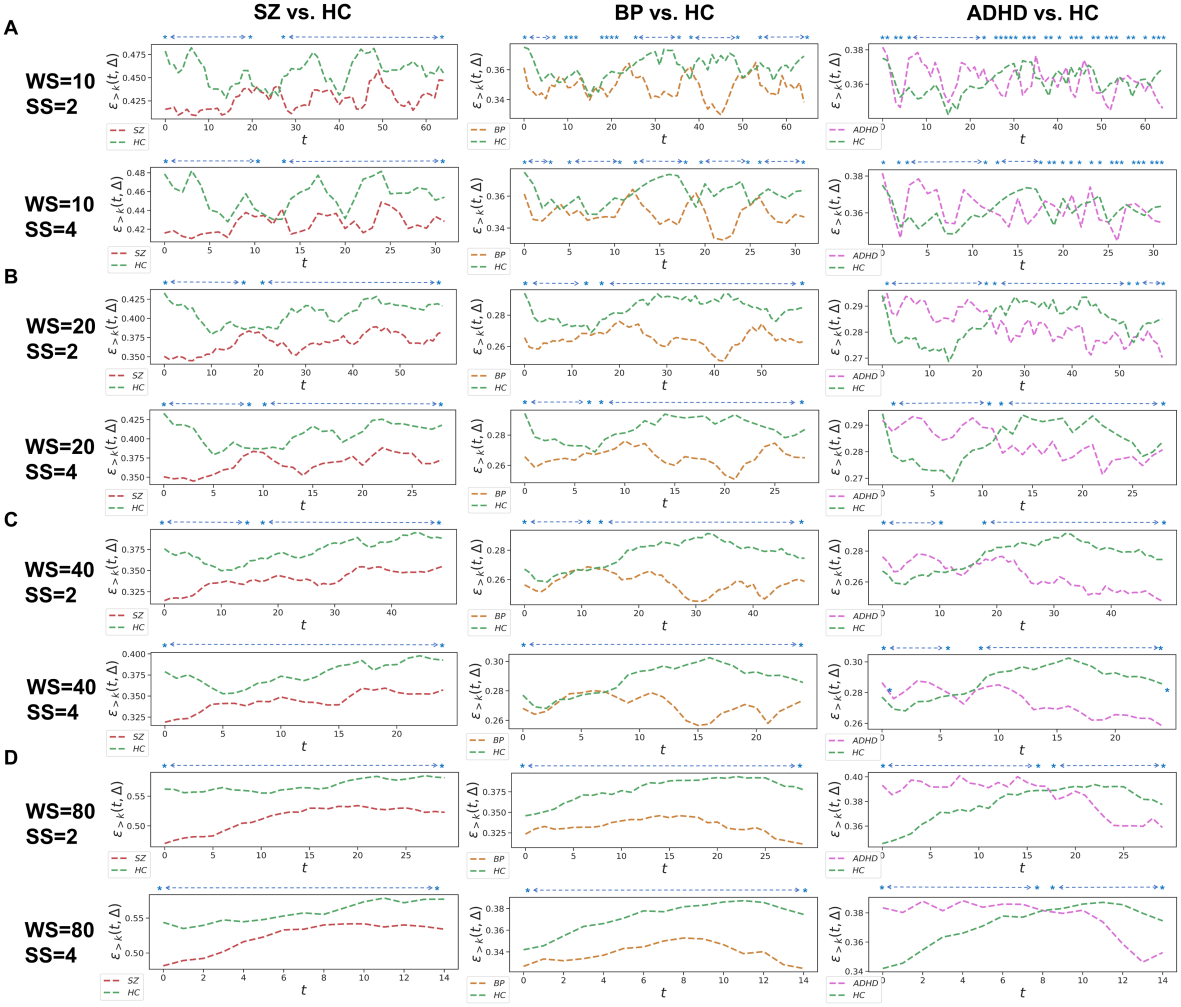
Whole-brain between-group TRC analyses of major psychiatric groups vs. corresponding HC groups on overlapped sliding window methods. The subgraph (A-D) are: (A) window size = 10 TR, sliding step = 2 TR; window size = 10 TR, sliding step = 4 TR; (B) window size = 20 TR, sliding step = 2 TR; window size = 20 TR, sliding step = 4 TR; (C) window size = 40 TR, sliding step = 2 TR; window size = 40 TR, sliding step = 4 TR; (D) window size = 80 TR, sliding step = 2 TR; window size = 80 TR, sliding step = 4 TR. All of the asterisks (*) denote significant between-group differences.

For overlapped sliding windows, we evaluated the following parameter settings (all adopting sparsity threshold R≥0.4): a. window size = 10 TR, sliding step = 2 TR; b. window size = 10 TR, sliding step = 4 TR; c. window size = 20 TR, sliding step = 2 TR; d. window size = 20 TR, sliding step = 4 TR; e. window size = 40 TR, sliding step = 2 TR; f. window size = 40 TR, sliding step = 4 TR; g. window size = 80 TR, sliding step = 2 TR; h. window size = 80 TR, sliding step = 4 TR. In our initial experiments, we have already validated that the impact of window size, sliding step and threshold R on TRC (also see Figures 1, 2) are significantly smaller relative to the Delta (Δ) itself. Therefore, to detect the subtle heterogeneity of these major psychiatric groups in stably maintaining the connections between the nodes (brain regions) of $S_{>k}$, we also performed whole-brain-scale TRC analyses with the altered duration Delta (Δ) from Delta = 1 to Delta = 8, based on the non-overlapping sliding window method with window size = 10 TR, sliding step = 10 TR, and sparsity threshold R≥0.4. A larger duration Δ denotes the connections are maintained for more fMRI timepoints.

**Figure 3:**
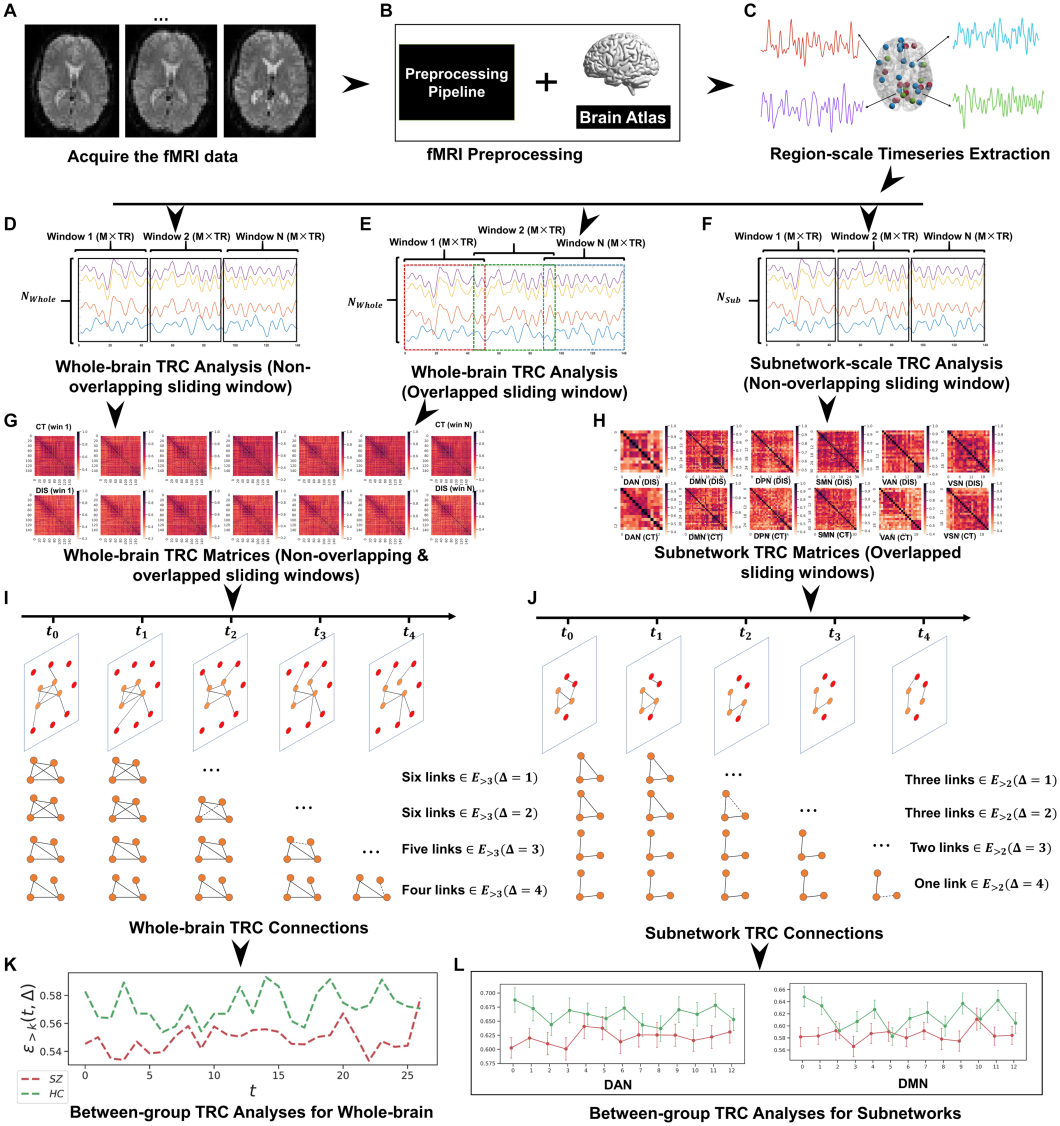
The whole framework performed in this study, which includes: **(A)** fMRI data acquisition; **(B)** fMRI data preprocessing based on Dosenbach’s 160 atlas; **(C)** region-scale timeseries extraction; **(D)** whole-brain TRC analysis (non-overlapping sliding window); **(E)** whole-brain TRC analysis (overlapped sliding window); **(F)** subnetwork-scale TRC analysis (non-overlapping sliding window); **(G)** whole-brain TRC matrices (including non-overlapping & overlapped sliding windows); **(H)** subnetwork TRC matrices (overlapped sliding windows); **(I)** whole-brain TRC connections; **(J)** subnetwork TRC connections; **(K)** between-group TRC analyses for whole-brain; and **(L)** between-group TRC analyses for subnetworks.

Since the results in non-overlapping sliding window strategies and overlapped sliding window strategies in whole-brain-scale TRC analyses display strong patterns in the consistency of results (see Figures 1, 2), the subnetwork-scale TRC analyses were directly performed on non-overlapping sliding window strategies (window size = 10 TR, sliding step = 10 TR) adopting the sparsity threshold from R≥0.2to R≥0.8. Our purpose here is mainly to avoid excessive display of redundant results. The basic flow of this study is depicted in Figure 3. Please note that in scenarios involving multiple comparisons, this study uniformly adopts FDR=0.05 as the multiple comparison correction threshold.

## 3. Results

### 3.1. Whole-brain TRC analysis of major psychiatric groups vs. HC group using non-overlapping sliding window methods

Utilizing a window size of 10 TR, a sliding step of 10 TR, and sparsity threshold of R≥0.2, we constructed group-scale average FC matrices for non-overlapping sliding windows for the COBRE (i.e., SZ and HC groups) and CNP (ADHD, BD and HC groups) databases (see in the Supplementary Figure S1). Based on these sliding windows, we computed whole-brain TRC coefficients for major psychiatric groups relative to their corresponding HC groups (Figure 1). Notably, differences in whole-brain TRC coefficients between major psychiatric groups and HC groups remain consistent for *R* values ranging from 0.2 to 0.8. Specifically, SZ and BD groups show significantly reduced average whole-brain TRC coefficients compared to their respective HC groups in most windows, indicating impaired TRC organizations. In contrast, TRC relationships between ADHD and HC groups are more complex, with whole-brain TRC coefficients significantly differing only in specific sliding windows.

To verify whether the TRC would be a powerful biomarker to identify psychiatric disorders, we performed subject-scale classification experiments for each major psychiatric group with their corresponding HC group. Table 3 displays all of the quantitative classification metrics based on an AdaBoost ensemble classifier (31) with 3-fold cross validation protocol (2 folds for training and 1 fold for test in each experiment). This protocol has been repeatedly performed for 10 times to obtain the mean results. In order to ensure the adequacy of the experiments, we conducted comparison experiments for the sparsity threshold for *R* ranging from 0.2 to 0.8. According to Table 3, we can easily note that the ability to recognize SZ (best ACC = 0.868 ± 0.07) using TRC is significantly stronger compared to ADHD (best ACC = 0.709 ± 0.10) or BD (best ACC=0.735±0.03). Additionally, the classification performance is better when *R* is approaching 0.2 rather than 0.8, which indicates that excessive network sparsity should be avoided when conducting TRC-based classification. These findings are consistent with the between-group difference results presented in Figure 1. However, we should also note that compared to the COBRE database, the CNP database has a more biased sample distribution, which may be one of the reasons for the worse classification results for ADHD and BD.

**Table 3:** Subject-scale classification performance of the three major psychiatric groups vs. their corresponding HC groups using TRC with non-overlapping sliding window methods.

Groups	ACC	SEN	SPE	PPV	NPV
SZ vs. HC (R = 0.2)	0.868±0.07	0.872±0.09	0.960±0.05	0.883±0.03	0.790±0.07
SZ vs. HC (R = 0.4)	0.859±0.04	0.835± 0.07	0.956±0.04	0.876±0.09	0.777±0.03
SZ vs. HC (R = 0.6)	0.844±0.01	0.822±0.04	0.947±0.03	0.850±0.10	0.762±0.11
SZ vs. HC (R = 0.8)	0.824±0.10	0.806±0.13	0.928±0.10	0.875±0.17	0.753±0.05
ADHD vs. HC (R = 0.2)	0.709±0.10	0.695±0.28	0.810±0.22	0.767±0.22	0.730±0.18
ADHD vs. HC (R = 0.4)	0.701±0.09	0.688± 0.22	0.783±0.32	0.742±0.35	0.718±0.25
ADHD vs. HC (R = 0.6)	0.682±0.13	0.665±0.34	0.754±0.39	0.722±0.48	0.689±0.49
ADHD vs. HC (R = 0.8)	0.643±0.35	0.632±0.49	0.701±0.29	0.673±0.50	0.630±0.38
BD vs. HC (R = 0.2)	0.735±0.03	0.724±0.08	0.877±0.11	0.852±0.08	0.767±0.05
BD vs. HC (R = 0.4)	0.718±0.08	0.712±0.09	0.838±0.12	0.832±0.12	0.758±0.02
BD vs. HC (R = 0.6)	0.691±0.12	0.681±0.12	0.810±0.23	0.797±0.45	0.736±0.34
BD vs. HC (R = 0.8)	0.676±0.49	0.622±0.69	0.754±0.47	0.760±0.83	0.624±0.61

ACC, accuracy; SEN, sensitivity; SPE, specificity; PPV, positive predictive value; NPV, negative predictive value. All of the values are denoted by Mean with SD.

### 3.2. Whole-brain TRC analysis of major psychiatric groups vs. HC group using overlapped sliding window methods

We further assessed TRC coefficients for major psychiatric groups relative to HC groups using overlapping sliding window methods, exploring various window sizes (e.g., *W* = 10, 20, 40, and 80 s) and sliding steps (e.g., 2s or 4s). Correlation matrices were constructed based on a sparsity threshold of R=0.4, yielding comparable results with R=0.2 and R=0.6. As shown in Figure 2, average TRC coefficients for SZ and BD groups were consistently lower than those for corresponding HC groups across all sliding windows (*p* < 0.05 in all paired *t*-tests). This pattern persisted across different window sizes (e.g., from WS=10s to WS=80s) and sliding steps (e.g., 2 s or 4 s). Similar to the non-overlapping sliding window methods, TRC coefficients in the between-group analysis of ADHD group vs. HC groups were complex and varied across different sliding windows.

### 3.3. Whole-brain TRC analysis of major psychiatric groups vs. HC group using different duration

**Figure 4:**
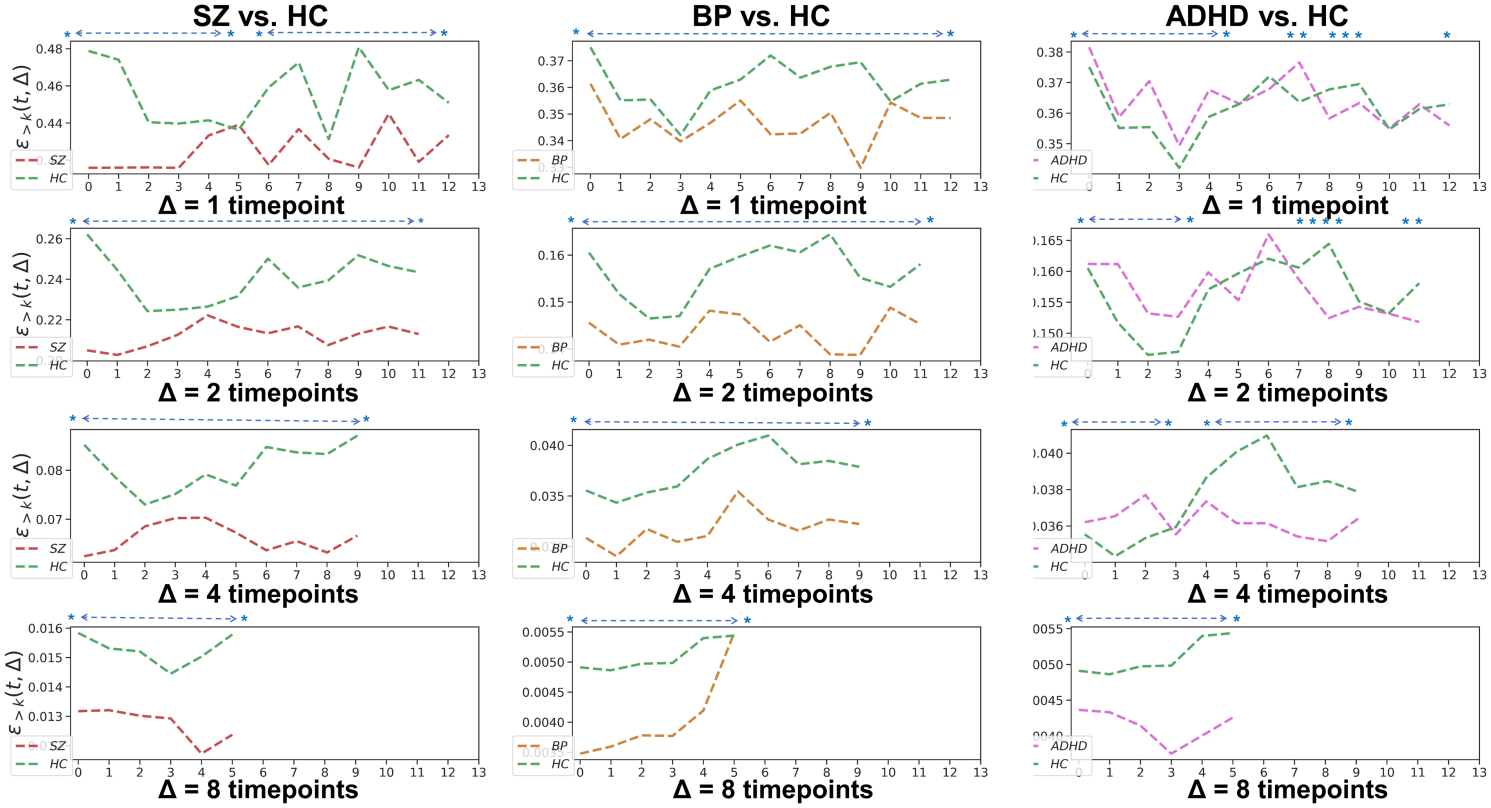
Whole-brain between-group TRC coefficients of subjects with mental disorders vs. corresponding HC subjects with different duration Δs. All of the asterisks (*) denote significant between-group differences.

To examine the homogeneity and heterogeneity of the three psychiatric disorders, we calculated TRC coefficients based on different duration (we denote it as Δ in figures). Figure 4 displays TRC coefficients for participants with psychiatric disorders relative to their corresponding HCs. Larger Δ values represent greater stability in the density of connected edges over time. Both SZ and BD groups consistently demonstrated smaller whole-brain average TRC coefficients than their corresponding HC groups (value of *p* < 0.05), with these patterns persisting from Δ = 1 to Δ = 8. In contrast, significantly smaller whole-brain TRC coefficients for the ADHD group compared to HC group were only observed for Δ = 8 and not for other smaller Δ values. These results suggest the underlying mechanisms of ADHD differ from those of SZ and BD, with reduced TRC coefficients only evident in longer durations.

### 3.4. Subnetwork-scale TRC analysis of major psychiatric groups vs. HC groups using non-overlapping sliding window methods

**Figure 5:**
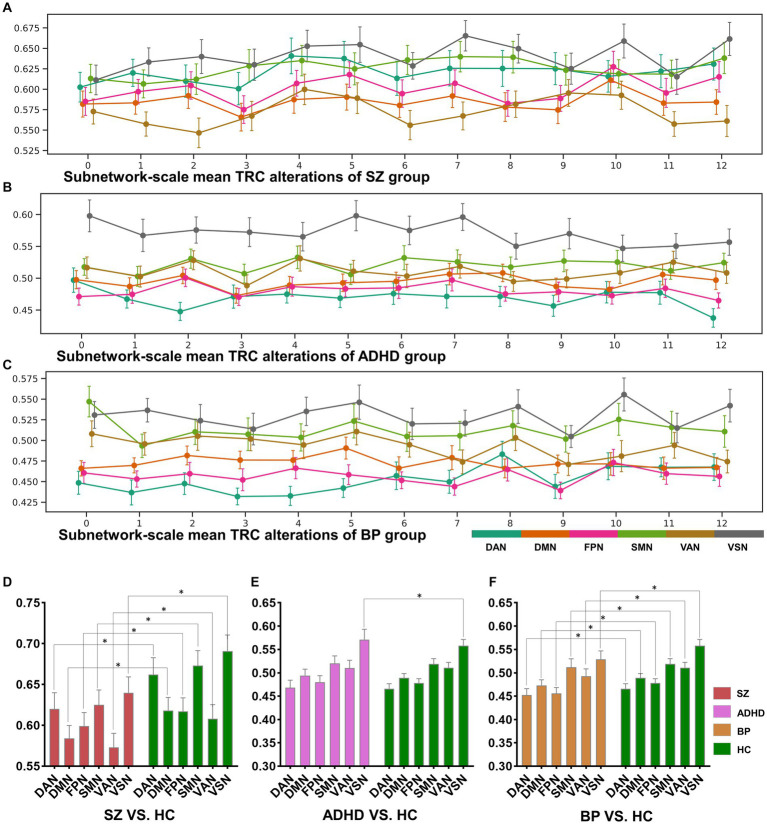
The sorts of subnetwork-scale mean TRCs for major psychiatric groups **(A–C)** and the corresponding between-group quantitative comparison results **(D–F)**. All of the asterisks (*) denote significant between-group differences.

In addition to whole-brain scale TRC analyses, we conducted subnetwork-scale TRC analyses to identify unique TRC alteration patterns in specific major psychiatric groups at a finer scale. For clarity, we illustrated the subnetwork-scale correlation matrices for the six subnetworks at non-overlapping window 1 and 14 (based on a window size = 10 TR and a sliding step of 10 TR) for the COBRE database in Figure 5. The sizes of FC matrices for DAN, DMN, FPN, SMN, VAN, and VSN were 14×14, 36×36, 28×28, 32×32, 21×21, and 22×22, respectively.

**Figure 6:**
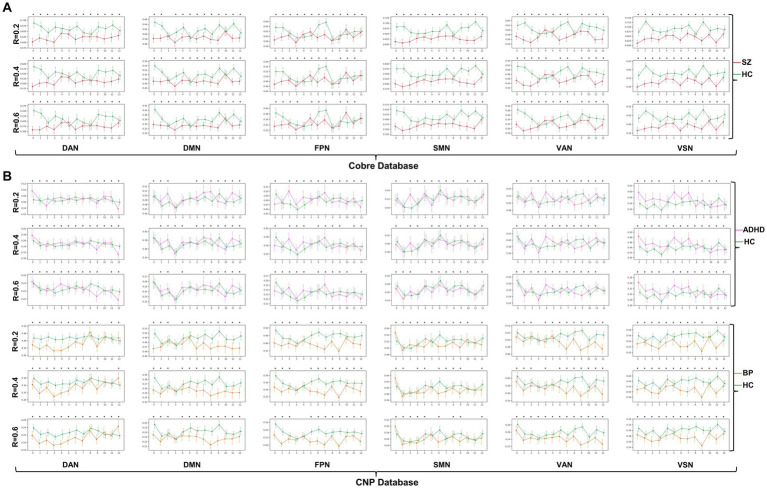
Dynamic change patterns of all subnetwork-scale TRC coefficients for **(A)** COBRE database and **(B)** CNP database. All of the asterisks (*) denote significant between-group differences.

The dynamic change patterns of all subnetwork-scale TRC coefficients for COBRE database and CNP database were also displayed in Figure 6. To ensure the stability of the subnetwork-scale TRC results, we conducted experiments using subnetwork-scale correlation matrices with three sparsity thresholds ranging from R=0.2 to R=0.6. Specifically, the SZ group exhibited significantly decreased TRC coefficients compared to the HC group for most sliding windows in DAN, DMN, SMN, VAN, and VSN, except for FPN; the ADHD group displayed fluctuating in TRC coefficients for single sliding windows compared to HC group in all subnetworks. However, the BD group showed significantly decreased TRC coefficients compared to the HC group for most sliding windows in DAN, DMN, FPN, VAN, and VSN, with a few exceptions in the SMN. These results indicate that the pathological patterns of the SZ group and the BD group are more similar to each other than the HC group in terms of subnetwork-scale TRC differences.

We further illustrated the subnetwork-scale mean TRC alterations for SZ, ADHD and BD groups in Figure 5, which help to demonstrate the overall trends of subnetwork-scale TRC coefficients for major psychiatric groups relative to corresponding HC groups. The top three subnetworks with the highest TRC coefficients were VSN (0.6406±0.1624), SMN (0.6257±0.1545), and DAN (0.6208±0.1683) for the SZ group; VSN (0.5709±0.1400), SMN (0.5201±0.1014), and VAN (0.5104±0.1061) for the ADHD group; and VSN (0.5297±0.1210), SMN (0.5129±0.1200), and VAN (0.4930±0.1037) for the BD group. Results indicate that all major psychiatric groups had significantly higher TRC coefficients in VSN and SMN than their corresponding HC groups (p<0.05 for *t*-tests). Quantitative comparisons can be found in Figures 5D–F, which reveal that both the SZ and BD groups exhibited significantly decreased average TRC coefficients in all six subnetworks compared to their corresponding HC groups (value of *p* < 0.01). However, for the ADHD group, only the TRC coefficients of VSN were significantly higher relative to HC group (value of *p* < 0.05).

## 4. Discussion

In this study, we present a novel TRC analysis approach to uncover the altered brain network patterns in ADHD, BD and SZ at both whole-brain and subnetwork scales. We considered various parameters such as network sparsity, sliding window strategies, window length, window step, and TRC coefficient duration. Our findings suggest that TRC coefficients may serve as effective biomarker to differentiate major psychiatric groups with similar phenotypes, and can complement the dynamic properties absent in existing graph theory metrics, particularly in the context of mental disorders.

At the whole-brain scale, we observed that SZ and BD groups displayed significantly decreased average whole-brain TRC coefficients in comparison to their corresponding HC groups across most windows, reflecting impaired TRC organizations. These findings were consistent across different network sparsity levels, sliding window strategies, window lengths, window steps, and TRC coefficient durations. In contrast, the ADHD group exhibited reduced TRC coefficients primarily in longer durations, indicating a fundamentally different TRC mechanism compared to SZ and BD groups (32). The results of subject-scale classification experiments indicate that TRC can serve as an important biomarker to distinguish psychiatric patients from the corresponding HCs. However, when the sample distribution is biased (e.g., ADHD and BD subjects in CNP database), the classification performance may significantly degrade. Collectively, the above results suggest that the three major psychiatric disorders are characterized by a diminished capacity for dynamic information processing, reflecting distinct cognitive processes.

At the subnetwork scale, we found that the SZ group exhibited significantly decreased TRC coefficients compared to the HC group across most sliding windows in DAN, DMN, SMN, VAN, and VSN, with the exception of FPN. This suggests that the SZ group’s brain network is less interconnected in a simultaneous and stable manner, indicating instability and fragility. The BD group, on the other hand, displayed significantly decreased TRC coefficients compared to the HC group across most sliding windows in DAN, DMN, FPN, VAN, and VSN, with a few exceptions in SMN. The presence of highly connected TRCs implies efficient information integration across various brain regions; therefore, significantly decreased TRC coefficients in BD individuals may critically impair the exchange of information between subnetworks and the coordination of cognitive processes (33, 34). Our results highlight the potential of subnetwork-scale TRC coefficients in FPN and SMN as useful biomarkers to differentiate SZ from the BD groups. Moreover, subnetwork-scale TRC coefficients for the ADHD group were also distinguishable, displaying significantly increased TRC coefficients in VSN compared to the HC group, a marked contrast to the decreased subnetwork-scale TRC coefficients observed in most subnetworks for SZ and BD groups.

Our findings align well with previous studies investigating FC in these disorders. For instance, prior SZ studies reported decreased FCs in brain regions primarily located in the DAN (35), FPN (36, 37), and SMN (38); BD studies found aberrant FCs in the DMN (39), FPN (40), and VAN (41); and ADHD studies identified hyperactivity in the VSN (32, 42). If the previous research results are compared to “pictures,” the results of this study are more like the “videos” corresponding to these pictures, which may own more information. For example, in the “picture” direction, both the SZ and BD groups displayed significantly decreased average TRC coefficients in all of the six subnetworks than their corresponding HC groups (see Figures 5D–F), but the dynamic change patterns (in the “video” direction) of TRC coefficients are different for SZ and BD groups: SZ group showed persistently decreased TRC coefficients in SMN and VSN, while the BD group showed persistently decreased TRC coefficients only in VSN (see Figures 5A,B). Also, in the “picture” direction, ADHD group showed significantly increased average TRC coefficients in VSN (see Figure 5E), but as for the dynamic change patterns (in the “video” direction), the ADHD group was usually indistinguishable from the HC group (see Figure 5B). All these evidences have shown the complementarity of TRC results to previous studies. Compared to these previous results, our study offers a more dynamic and informative perspective on brain network alterations in mental disorders. The complementarity of TRC results to previous research is evident in both static and dynamic patterns.

Altered subnetwork-scale TRC coefficients may accurately reflect disorder-specific impairments in neural circuit mechanisms. For example, previous studies has shown that decreased connections in VSN are common features in both SZ and BD groups, and that individuals with BD may compensate for disrupted VSN connectivity while those with SZ do not (43). Our results show that mean TRC gaps between BD and HC groups are 0.028±0.018, which are significantly smaller than mean TRC gaps between SZ and HC groups (0.051±0.028). Obviously, smaller TRC gaps are easier to be compensated. Similar patterns can also be found in the SMN of SZ group and BD group. Results in Figures 5A–C show that all SZ, ADHD and BD groups have the highest TRCs in VSN, which points out the neural circuit bias of visual processing ability in mental disorders (32, 43). Our findings support previous observations and reveal complementary information on the dynamic and static aspects of subnetwork-scale TRCs, underscoring the importance of including both in analyses of mental disorders.

Despite its contributions, this study has certain limitations that should be considered when interpreting the results. First, although the TRC analysis workflow has been performed at both whole-brain and subnetwork scales across different parameter settings, there are many properties of TRC still under explore. For example, in future study, the TRC analysis could be further applied to regional scale to display the hub distribution patterns (e.g., hub nodes and feeder nodes). Second, due to the limited data, we fail to correlate TRC with some clinical or cognitive scales, thus impairing the clinical interpretability. Third, the TRC analysis workflow employed in this study was mainly based on the linear correlation that one brain region may influence another, ignoring the inherently nonlinear properties of fMRI signals, future studies may benefit from examining the differences in non-linear relationships among rich-club regions.

## 5. Conclusion

We present the TRC analysis workflow as a comprehensive method to uncover altered rich-club patterns in major psychiatric conditions at both whole-brain and subnetwork levels. Our results reveal that SZ and BD groups exhibit significantly decreased TRC coefficients compared to their corresponding HC groups at the whole-brain scale and in most subnetworks. Conversely, the ADHD group exhibits reduced TRC coefficients in longer durations, rather than shorter duration, which significantly differs from the SZ and BD groups. Our findings highlight the potential of TRC coefficients as effective biomarkers to distinguish between psychiatric groups with similar phenotypes, and emphasize the importance of considering both static and dynamic aspects of subnetwork-scale TRCs in the study of mental disorders.

## Data availability statement

The original contributions presented in the study are included in the article/Supplementary material, further inquiries can be directed to the corresponding authors.

## Ethics statement

The imaging data and phenotypic information was collected and shared by the Mind Research Network and the University of New Mexico funded by a National Institute of Health Center of Biomedical Research Excellence grant 1P20RR021938-01A2 and the Consortium for Neuropsychiatric Phenomics (NIH Roadmap for Medical Research grants UL1-DE019580, RL1MH083268, RL1MH083269, RL1DA024853, RL1MH083270, RL1LM009833, PL1MH083271, and PL1NS062410). The studies were conducted in accordance with the local legislation and institutional requirements. Written informed consent for participation was not required from the participants or the participants’ legal guardians/next of kin in accordance with the national legislation and institutional requirements.

## Author contributions

MN and HG contributed to conception and statistical analysis of the study. YF wrote the first draft of the manuscript, performed validation, and conducted the whole project. ZZ revised the first draft of the manuscript and performed validation. All authors contributed to the article and approved the submitted version.

## Funding

This study was supported by the Zhejiang Xinmiao Talents Program (2023R401197), the Innovation Fund of the Department of Education of Gansu Province (2022B-023), and the Fund of The First Hospital of Lanzhou University. China (Ldyyyn2021-73).

## Conflict of interest

The authors declare that the research was conducted in the absence of any commercial or financial relationships that could be construed as a potential conflict of interest.

## Publisher’s note

All claims expressed in this article are solely those of the authors and do not necessarily represent those of their affiliated organizations, or those of the publisher, the editors and the reviewers. Any product that may be evaluated in this article, or claim that may be made by its manufacturer, is not guaranteed or endorsed by the publisher.

## Footnotes

^1^ http://fcon\_1000.projects.nitrc.org/indi/retro/cobre.html

^2^ https://openneuro.org/
