# Supplementary material for: Abnormal temporal variability of rich-club organization in three major psychiatric conditions
Source: Front Psychiatry. 2023 Aug 31;14:1226143. doi: 10.3389/fpsyt.2023.1226143 (PMC10500439; doi:10.3389/fpsyt.2023.1226143)

Supplementary Material for: Abnormal temporal variability of rich-club organization in three major psychiatric conditions

Meng Niu^1,2,3^, Hanning Guo^4^, Zhe Zhang^5, 6*^ and Yu Fu^7*^

^1^Department of Radiology, The First Hospital of Lanzhou University, Lanzhou, China.

^2^Intelligent Imaging Medical Engineering Research Center of Gansu Province, Lanzhou, China.

^3^Accurate Image Collaborative Innovation International Science and Technology Cooperation Base of Gansu Province, Lanzhou, China.

^4^Institute of Neuroscience and Medicine, Medical Imaging Physics (INM-4), Forschungszentrum Jülich, Jülich, Germany.

^5^School of Physics, Hangzhou Normal University, Hangzhou, China.

^6^Institute of Brain Science, Hangzhou Normal University, Hangzhou, China.

^7^College of Information Science & Electronic Engineering, Zhejiang University, Hangzhou, China.

^*^ Correspondence: Zhe Zhang [(zhangz@hznu.edu.cn)](mailto:(yusun@zju.edu.cn)) or Yu Fu [(yufu1994@zju.edu.cn)](mailto:(yufu1994@zju.edu.cn))

**Fig. S1.** Visualization of group-scale average non-overlapping sliding windows for (A) COBRE database and (B) CNP database.


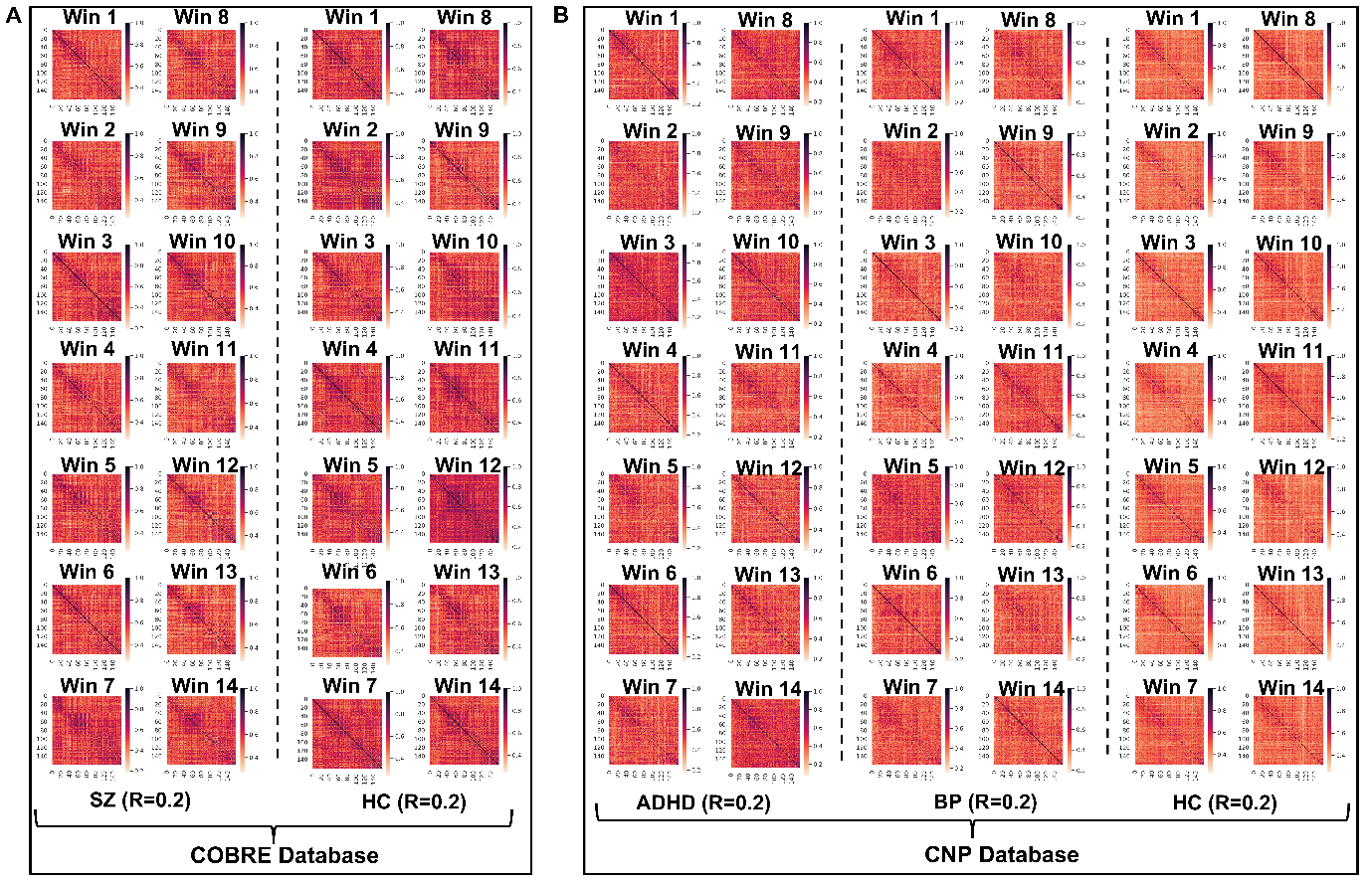

Supplement: Supplementary file 1 [file Data_Sheet_1.docx]
